# Neuroscience: Implications for Treatment

**Published:** 1997

**Authors:** Ismene Petrakis, John Krystal

**Affiliations:** Ismene Petrakis, M.D., is an assistant professor and John Krystal, M.D., is an associate professor in the Department of Psychiatry, Yale University, New Haven, Connecticut

**Keywords:** drug therapy, AOD dependence, anti alcohol craving agents, reinforcement, naltrexone, calcium acetylhomotaurinate, serotonin uptake inhibitors, anxiety state, emotional and psychiatric depression, intelligence and ability, alcohol relapse prevention agents, neurotransmitter receptors, literature review

## Abstract

Medications can be used to treat alcoholism by decreasing craving for alcohol or by blocking the development of processes that lead to addiction. Medications also can enable alcoholic patients to participate more effectively in treatment by decreasing psychiatric symptoms associated with alcoholism or by improving alcohol-related intellectual impairment. Many promising medications for treating alcoholism appear to play a role in normalizing communication among nerve cells. Such medications include naltrexone and acamprosate, which help prevent relapse in patients undergoing psychotherapy for alcoholism. Medications that affect the neurotransmitter serotonin, however, may alleviate symptoms of anxiety and depression in alcoholics without affecting the alcoholism itself.

Recent advances in neuroscience have stimulated the search for medications to treat alcoholism. Medications development relies on both preclinical and clinical investigations. Preclinical research studies the effects of experimental chemicals on the physiology and behavior of laboratory animals or on the function of cells grown in culture. Results of such research provide the basis for clinical studies (i.e., trials) of specific medications in humans.

Preclinical research has established that alcohol affects brain function by interfering with communication among nerve cells (i.e., neurons). Researchers have identified specific neural communication systems that contribute to the rewarding effects of alcohol consumption, thereby promoting alcoholism. Preexisting defects in such systems also may underlie vulnerability to excessive alcohol consumption. Neurons communicate by synthesizing and releasing chemicals called neurotransmitters, which bind to specific receptor^1^ molecules on the surface of other neurons. Scientists can investigate this process using natural or synthetic chemicals that affect specific neurotransmitter systems. These experimental chemicals can influence neurotransmission by directly affecting the synthesis or release of a neurotransmitter, by affecting the rate of removal of a neurotransmitter after its release, or by binding to a receptor and either activating or inactivating it. The linking of these neurotransmitter systems to behavior and even to psychiatric disorders has greatly influenced clinical research and has contributed to the development of novel pharmacotherapies for a number of psychiatric disorders, including alcoholism.

Clinically, medications can be used to treat alcoholism or its effects by (1) decreasing symptoms associated with the initial stages of recovery, such as anxiety or depression; (2) decreasing a person’s desire to consume alcohol; (3) blocking a component of the addictive process (i.e., alcohol’s reinforcing effects); (4) treating psychiatric disorders that might coexist with alcoholism; and (5) improving alcohol-related intellectual impairment (Meyer 1989). Although some of these treatment goals are not directed against alcoholism per se, amelioration of disorders associated with alcoholism may enable the patient to participate more effectively in his or her treatment.

This article discusses medications whose effects fall into one or more of the first four categories, including naltrexone (ReVia^®^), selective serotonin reuptake inhibitors (SSRI’s), and acamprosate.

## The Opioid Antagonists: Naltrexone

Endogenous opioids are neurotransmitters whose actions in the brain are similar to that of morphine or heroin (which do not occur naturally in the body). Behavioral and pharmacological data in animals and humans suggest a link between the endogenous opioid system and alcohol consumption (reviewed in Froehlich and Li 1994; Schuckit 1996). For example, administration of alcohol induces the release of endogenous opioids in the hypothalamus, a region of the brain involved in the regulation of various physiological states, including mood, sleep, and appetite. This effect is especially pronounced in animals bred for high alcohol preference. Overall alcohol consumption is decreased by the opioid antagonists naltrexone and naloxone.

These findings suggest that the opioid system may play a role in regulating drinking behavior; however, the nature of that role is uncertain. According to the opioid surfeit hypothesis, a biological predisposition to excessive alcohol consumption may result from overactivity of the endogenous opioid system. Conversely, the opioid deficit hypothesis states that a biological propensity to consume alcohol may result from underactivity of the opioid system. More recently, scientists have proposed the opioid response hypothesis, which states that although baseline levels of opioid activity do not affect alcohol consumption, alcohol use leads to an increase in endogenous opioid activity, which then promotes alcohol’s reinforcing effects (reviewed in Swift 1995; Froehlich and Li 1994). This hypothesis is supported in part by the demonstration that opioids stimulate the release of dopamine, a neurotransmitter implicated in reinforcement. Therefore, researchers have hypothesized that a medication that blocks opioid activity may block the reinforcing aspects of alcohol (Swift 1995).

Naltrexone—a medication used to treat heroin addiction—blocks opioid receptors by competing for neurotransmitter binding sites. Two clinical trials established the efficacy of naltrexone in treating alcoholism. In a 12-week, double-blind, placebo-controlled trial, Volpicelli and colleagues (1992) found that only 23 percent of alcoholic subjects who were administered naltrexone relapsed to heavy drinking, compared with 54 percent of subjects taking a placebo. Treatment with naltrexone was associated with a decreased number of drinks per drinking day and a lower relapse rate among subjects who sampled alcohol after achieving abstinence. In another large, double-blind, placebo-controlled clinical trial (O’Malley et al. 1992), both naltrexone- and placebo-treated subjects were randomly assigned to one of two psychosocial treatments. One-half of each group of patients (i.e., the placebo-treated subjects and the naltrexone-treated subjects) received coping-skills training, a form of psychotherapy that teaches patients how to handle problems without resorting to alcohol. The remaining subjects received supportive counseling that emphasized abstinence. Naltrexone-treated subjects exhibited a higher rate of abstinence and better employment records than the placebo-treated patients, regardless of the type of psychotherapy provided. Psychotherapy interacted with the effect of medication, however. Overall, the highest rate of abstinence occurred in patients treated with both naltrexone and supportive therapy. Among subjects who sampled alcohol after abstinence, those receiving naltrexone and coping-skills treatment combined were less likely than other subjects to experience a relapse to heavy drinking. Based in part on these findings, the U.S. Food and Drug Administration approved naltrexone for the treatment of alcohol dependence.

Naltrexone’s mode of action remains uncertain. In the Volpicelli study, although treatment with naltrexone did not significantly reduce the percentage of subjects who sampled alcohol during the study, it significantly reduced the rate of return to excessive drinking (Volpicelli et al. 1995). Subjects who were administered naltrexone reported less craving than did those on placebo. In addition, compared with the patients taking placebo, subjects who consumed alcohol while on naltrexone reported significantly less of a “high” while drinking. In the O’Malley study, retrospective reports of craving were lower for the naltrexone-treated group than for the placebo-treated group. Among subjects who resumed drinking, those on naltrexone reported that they stopped drinking for reasons consistent with decreased incentive to drink (O’Malley 1995). In a separate laboratory study evaluating the effect of naltrexone on subsequent alcohol consumption, participants reported significantly fewer subjective effects of alcohol than those pretreated with placebo (Davidson et al. 1996). These and other studies suggest that naltrexone reduces craving for alcohol, loss of control over drinking, and some of alcohol’s subjective pleasurable effects.

Despite the promise of naltrexone, some questions have emerged that were not predicted by preclinical studies. For example, a small percentage of patients taking naltrexone develop nausea, which is thought to reflect an exacerbation of alcohol withdrawal symptoms (O’Malley et al. 1992). This raises the possibility that the timing of initiation of pharmacotherapy may influence the clinical outcome. If naltrexone precipitates symptoms of withdrawal, it may not be tolerated by patients if it is initiated during the early states of recovery. Another factor requiring additional study is the appropriate matching of psychotherapy to a course of naltrexone treatment—in both major clinical trials previously discussed (Volpicelli et al. 1992; O’Malley et al. 1992), naltrexone was administered as an adjunct to psychosocial treatment. Naltrexone’s long-term efficacy and cost-effectiveness also are unknown.

## Medications That Affect Serotonin Function

The neurotransmitter serotonin (5-HT) affects multiple actions in the brain, including the regulation of mood states, appetite, and sleep. This diversity of function has been linked to the existence of several specific serotonin receptor subtypes as well as variability in the mechanisms whereby receptor activation is translated into neuronal function.

Preclinical studies suggest a relationship between serotonin function and alcohol consumption. For example, serotonin administration causes a decrease in alcohol consumption in animals selectively bred for alcohol preference as well as in animals that are genetically heterogeneous (reviewed in Naranjo et al. 1986). This effect may not be specific to alcohol, because an increase in the availability of brain serotonin also may decrease consumption of food and nonalcoholic liquids (reviewed in Sellers et al. 1992). Other studies suggest that serotonin helps regulate reinforcement, because disturbances in the serotonin system may selectively affect consumption of rewarding substances such as alcohol, other drugs, and sweet-tasting substances (Sellers et al. 1992).

Laboratory studies have found abnormalities in serotonin activity associated with alcohol use, alcoholism, and impulsivity (Sellers et al. 1992). These abnormalities are more prominent in type II alcoholics, a group characterized by early onset of drinking and significant detrimental social consequences related to alcoholism.

The exact nature of the relationship between serotonin and alcoholism is unknown. One theory suggests that alcoholics are naturally deficient in brain serotonin. According to this view, alcoholism may represent an attempt to increase brain serotonin levels. Another theory suggests that serotonin either directly influences the reinforcing effects of alcohol and other drugs or exerts an indirect influence, through an effect on the neurotransmitter dopamine. A third suggestion is that low levels of serotonin lead to impulsive behavior, including an inability to modulate alcohol intake. Serotonergic abnormalities also may contribute to anxiety, potentially leading to the “self-medication” of anxiety symptoms with alcohol. Finally, serotonergic activity may affect general appetitive behaviors (reviewed in Schuckit 1996).

Among the medications evaluated for alcoholism treatment are several selective serotonin reuptake inhibitors (SSRI’s) prescribed to treat depressive disorders. SSRI’s increase the activity of serotonin by preventing its reabsorption into the neuron that released it. In several clinical trials, Naranjo and colleagues (1984, 1987, and 1990) evaluated the effects of three different SSRI’s—zimelidine, fluoxetine, and citalopram—on alcohol consumption by heavy drinkers. Although each medication decreased alcohol consumption, its effect was small. Two more recent clinical trials showed no effect of fluoxetine or citalopram on alcohol consumption by alcoholics. These studies suggest that SSRI’s have limited utility for treating alcoholism. However, some evidence suggests that they may benefit alcoholics who experience co-occurring depression (Cornelius et al. 1993).

Results of preclinical and clinical laboratory studies may help explain these disappointing results. First, if altered levels of serotonin underlie alcoholism, serotonin agonists might be more effective in reducing alcohol consumption than would reuptake inhibitors, which rely on adequate pre-existing levels of serotonin for their action. Second, different aspects of alcohol consumption and alcoholism may involve different serotonin receptor subtypes. Medications targeted at specific receptor subtypes may affect alcohol consumption more effectively than a medication like fluoxetine, which increases serotonin activity nonspecifically.

Buspirone (Buspar^®^) is an antianxiety medication that affects the serotonin system. Although buspirone decreases alcohol consumption in animals, its clinical application in human alcoholics is unclear. Several small clinical trials found that buspirone decreased symptoms of anxiety in alcoholics but not alcohol consumption. In a 12-week clinical trial evaluating the use of buspirone in combination with coping-skills treatment in anxious alcoholics, patients treated with buspirone showed a trend toward a lower number of drinking days and a lower number of drinks per drinking day (Kranzler et al. 1994). Overall, however, although buspirone consistently decreases alcohol consumption in laboratory animals, its effect in humans is small. The most promising use for this medication is in the treatment of alcoholics with coexisting anxiety disorders.

## Amino Acid Neurotransmitters

The amino acid glutamate is the most prevalent excitatory neurotransmitter in the brain, where it modulates arousal by making neurons more sensitive to further neurotransmission. Alcohol is a glutamate antagonist, blocking the NMDA receptor, a glutamate receptor subtype so named because it responds to the agonist *N*-methyl-d-aspartate (reviewed in Tsai et al. 1995). The NMDA receptor is involved in learning, memory, and seizure activity, functions known to be influenced by alcohol consumption.

In animal models, alcohol has been shown to manifest several of its behavioral effects (e.g., impaired motor skills and cognition) through the NMDA receptor. Studies in humans provide evidence that NMDA antagonists produce alcohol-like properties in humans and can modulate the effects of alcohol intoxication, including euphoria, a “high” feeling, and sedation. In addition, evidence suggests that alcohol’s reinforcing properties may be related in part to its effects on the NMDA receptor (reviewed in Tsai et al. 1995), possibly through glutamate’s influence on dopamine activity.

Upon long-term alcohol exposure, NMDA receptors undergo increased activity (i.e., up-regulation) to help compensate for alcohol’s continued antagonistic effect. At the end of a drinking bout, the now hyperactive glutamate system is no longer balanced by the presence of alcohol, leading to tremor, high blood pressure, sweating, and seizures (i.e., withdrawal syndrome). Administration of NMDA antagonists is one form of treatment that can suppress withdrawal-induced seizures in animals. Persistent alteration in NMDA receptor function in alcoholics may increase the risk of seizures and contribute to the development of alcohol-related organic brain disease. Thus, abnormalities of glutamate function may be involved in effects of alcoholism ranging from acute intoxication and addiction to long-term nervous system effects. This possibility suggests the recent hypothesis that alcoholism may be understood as a glutamate-related neuropsychiatric disorder (reviewed in Tsai et al. 1995).

These preclinical and clinical studies have suggested that glutamatergic medications may reduce alcohol consumption. The leading candidate in this area is acamprosate. Preclinical research suggests that acamprosate may block glutamate reuptake (Cammack et al. 1991). A series of placebo-controlled European studies have indicated that detoxified alcoholics treated with acamprosate were less likely to drop out of treatment and achieved higher rates of abstinence (Sass et al. 1996) than those on placebo. It has been hypothesized that acamprosate may help patients achieve abstinence at least in part by diminishing withdrawal symptoms. Additional clinical trials are required to answer questions raised about the use of acamprosate as a medication for alcoholism treatment, including the following: Does acamprosate decrease drinking in alcoholics? If so, for what population is it appropriate? Should it be paired with a psychosocial treatment, and if so, which one?

## Future Directions

Because alcoholism is a complex disorder, its treatment should be multifaceted. Effective treatment must target the extensive social, psychological, and medical complications of alcoholism. Medications can play various, complementary roles in alcoholism treatment. For example, acamprosate may decrease symptoms associated with early recovery, naltrexone may decrease the desire to drink or may block the reinforcing aspects of alcohol, and medications such as fluoxetine or buspirone may help in treating co-occurring psychiatric disorders. Clinicians can use medications to ease patients into psychosocial treatments or to enhance the effect of psychosocial treatments in decreasing alcohol consumption. The optimal clinical applicability for these medications has yet to be fully examined. Clinical and neuroscience research will continue to increase our understanding of the applications and limitations of proposed treatments while stimulating the development of new medications.

## Footnotes

1 For a definition of this and other technical terms, see central glossary, pp. 177–179.
